# A Phenomenon: What Are the Minuscule Grey Moths Abundant in the Dry Season in the Tropical Dry Forests of the Pacific Coast of Honduras? **

**DOI:** 10.3390/insects15090641

**Published:** 2024-08-26

**Authors:** Jonas R. Stonis, Andrius Remeikis, Arūnas Diškus, Viktorija Dobrynina, Svetlana Orlovskytė

**Affiliations:** State Research Institute Nature Research Centre, Akademijos g. 2, LT-08412 Vilnius, Lithuania; remeikis.andrew@gmail.com (A.R.); diskus.biotaxonomy@gmail.com (A.D.); viktorija.dobrynina@gamtc.lt (V.D.); s.orlovskyte@gmail.com (S.O.)

**Keywords:** *Acalyptris*, atypical characters, Central America, dry season, fauna, Lepidoptera, Nepticulidae, new species, pygmy moths, tropical dry forests

## Abstract

**Simple Summary:**

Understanding the diversity, distribution, and ecological roles of leaf-mining Lepidoptera across different biomes is both intriguing and important for advancing our knowledge of biodiversity and ecosystem functioning. Despite extensive studies on leaf-mining Nepticulidae in tropical environments, no one had previously explored trapping in completely dry deciduous forests, especially during the peak of the dry season when trees are bare and even grass is dried out. This paper reveals an unexpected and astonishing abundance of minuscule plant-mining Nepticulidae moths in such dry deciduous forests. Our study ecoregion, the tropical dry forests of Honduras, includes the Pacific coastal lowlands and premontane areas extending into low-altitude regions further inland, known for their rich biodiversity and high levels of endemism. In these tropical dry forests, we identified five species of pygmy moths belonging to the genus *Acalyptris* Meyrick, including three new species. These moths are characterized by their distinctive grey coloration and exceptionally small size, classified as “extremely small”. Despite their similarities, they exhibit significant differences in genital structures and molecular profiles, indicating distinct species groups. Our research also uncovered novel atypical morphological traits in Nepticulidae from this ecoregion. These findings highlight the unique and highly specific nature of the Nepticulidae fauna in tropical dry forests. A key question arises regarding the presence of Nepticulidae adults during the dry season: could they be mining plant bark instead of leaves? This paper aims to stimulate further exploration of micromoths in other tropical dry forests, which, despite their limited and fragmented distribution, are found not only in Central America but also in other regions worldwide.

**Abstract:**

Our investigation centered on the tropical dry forests along the Pacific coast of Honduras, aiming to elucidate the presence and abundance of minuscule grey moths during the dry season. Through specimen dissections and the taxonomic identification of the collected material, we have described three new species: *Acalyptris podenasi* sp. nov., *A. palpiformis* sp. nov., and *A. tortoris* sp. nov. Additionally, we documented two species previously known from neighboring countries, *A. lascuevella* Puplesis & Robinson and *A. basicornis* Remeikis & Stonis. The females of *A. lascuevella* were previously unknown and are documented here for the first time. Morphological examinations were complemented by DNA barcoding, particularly highlighting variation in *A. lascuevella*. The paper’s primary significance lies not only in the description of new species but also in uncovering their taxonomic, morphological, and molecular importance. We found that these species are unique and indicative of the previously unstudied dry forests as a distinct ecosystem. Our findings revealed several novel atypical morphological traits within the studied Nepticulidae, including unusually large signum cells in the female genitalia, a dorso-ventrally divided uncus, and asymmetrical valvae in the male genitalia. These discoveries underscore the morphological diversity of *Acalyptris* Meyrick and their significance in evolutionary biology. Consequently, the paper addresses a previously unknown phenomenon of the occurrence and astonishing abundance of minuscule plant-mining micromoths in dry deciduous forests during the peak of the dry season. We hope that this paper will encourage Lepidoptera taxonomists to explore micromoths in other tropical dry forests, which, while limited in distribution, hold global importance. The paper is extensively illustrated with photographs of *Acalyptris* adults and their genitalia, along with maps, habitats, and molecular phylogenetic trees.

## 1. Introduction

**Pygmy Moths.** Nepticulidae, commonly known as pygmy moths, represent a peculiar and distinct family of minuscule moths within the order Lepidoptera. Recent studies indicate that pygmy moths originated in the Early Cretaceous [1], making this family one of the basal phylogenetic lineages of extant Lepidoptera [2]. Despite being phylogenetically primitive, they exhibit remarkable specialization. They are among the smallest moths in the world, with forewing lengths in the record-small species ranging from just 1.13 to 1.3 mm and wingspans between 2.7 and 2.8 mm [3]. Nepticulidae are found worldwide, inhabiting various biomes, from tundra and temperate forests to subtropical deserts and rainforests, and they occur in a remarkably diverse range of terrestrial habitats, including anthropogenic ones.

The larvae of most Nepticulidae species are leaf miners, burrowing into leaves to feed and develop through all of their larval instars. Only some species induce galls or mine within stems, young bark, and buds (see pictorial guide by Stonis et al. [4]). Notably, the vast majority of Nepticulidae species are monophagous or oligophagous.

Research into pygmy moths not only enhances our understanding of biodiversity and phylogenetics but also has practical implications for agriculture, horticulture, and forestry. Their intriguing and yet uncovered diversity, along with their ecological and morphological adaptations, continue to be subjects of study and interest in the scientific community. The family has been extensively reviewed in comprehensive studies by Scoble [5], Johansson et al. [6], Puplesis [7], and Puplesis & Diškus [8]. Recent research by Stonis et al. [9] has particularly focused on Neotropical America, and we recommend their work for further exploration of this subject.

**Knowledge Gaps and Opportunities.** Currently, the family Nepticulidae comprises 1018 species worldwide [9,10,11], including the three new species described in the current paper.

Our investigation focused on the tropical dry forests along the Pacific coast of Honduras, Central America. While tropical dry forests occur in various regions worldwide, specific knowledge about the Nepticulidae from this biome has been notably scarce. The Nepticulidae fauna of Central America remains insufficiently and unevenly explored. In 2000, Rimantas Puplesis and Gaden S. Robinson reviewed the Nepticulidae of Belize [12], describing around 30 new species and reporting a few previously known species. This number was increased later with the description of 17 new species from Belize [13,14], Mexico [15,16], Guatemala [13,17,18], and Costa Rica [14,19]. The Nepticulidae of Central America were also reviewed in the comprehensive publication “Catalogue of *Acalyptris* of the Americas” [20], which provided an overview of 24 *Acalyptris* Meyrick species from tropical areas of Mexico, Belize, Guatemala, and Costa Rica, and the monograph “Neotropical Nepticulidae” [9]. In the latter publication, two previously documented but unnamed species from Belize and Guatemala were re-described and named. However, this is still very little compared to the anticipated actual diversity of Nepticulidae in this biodiversity-rich region. Until now, the Nepticulidae fauna in some Central American countries, like Honduras, remained totally unexplored.

Thus, while progress has been made in documenting Nepticulidae species in Belize and to a lesser extent in Guatemala, Mexico, and Costa Rica, there remains a clear opportunity and necessity for further research to enhance our understanding of the diversity, distribution, and ecological roles of Nepticulidae across Central America. Recognizing this gap, we conducted targeted fieldwork in 2023 in Honduras, marking the first focused study of Nepticulidae in this country.

**Tropical Dry Forests in Honduras.** Honduras features a predominantly mountainous terrain with lowlands situated along the coasts or nestled within river valleys. The country experiences a tropical climate and boasts a diverse natural landscape that includes various ecoregions [21,22]: tropical Atlantic moist forests, sometimes referred to as Atlantic humid forests (or, incorrectly, as rainforests), cloud forests, mangroves, savannas, montane forests, pine-oak forests (with *Pinus oocarpa* Schiede ex Schltdl. and *Quercus* spp., [23]), and tropical dry forests; the latter are often also referred to as Central American dry forests (e.g., [22,24]) or by other names (see [25]), including the term “tropophilous forest” [26]. 

The tropical dry forests, a distinct ecoregion within the Central American mixed forests bioregion [24], are considered one of the world’s most endangered biomes [27]. In Honduras, this ecoregion primarily covers the Pacific coastal lowlands and premontane areas, typically extending up to 800 m in elevation, and constitutes a relatively small portion of the country’s land area. However, significant expanses of these dry forests are also found in low-altitude regions further inland, including central and northern Honduras [28]. The tree canopy in these dry deciduous forests reaches heights of approximately 30 m and is characterized by delicate, compound leaves that are shed seasonally. The understory often features evergreen species, with thorny trees, woody lianas, and epiphytes being common [21,25,28]. 

This ecoregion is notably distinct due to its pronounced seasonal variations. For six to eight months of the year, rainfall is minimal, with a brief rainy season occurring only between July and September [21]. During the extended dry period, trees shed their leaves to conserve water—a survival strategy adopted by various species to endure prolonged water scarcity [28]. The tropical dry forests play a crucial role in the migration routes and life cycles of many species [21]. Although these forests are generally smaller in structure and simpler in composition compared to moist forests, they remain dense and remarkably species-rich [21,27,29]. This ecoregion is home to a significant percentage of endemic species [21,28]. Unfortunately, due to deforestation, migratory agriculture and agricultural burning [30], cattle ranching, and urban development, approximately 80% of this ecoregion has been converted into settlements, cattle ranches, and plantations [23,28].

**Study Objective and Significance.** During our fieldwork in the dry season within Honduras’ dry forests, we collected a substantial number of Nepticulidae. Among the collected specimens, extremely tiny, grey-colored moths were prevalent and abundant. Given that Nepticulidae are typically leaf miners, it was unusual to observe such high numbers of adults in forests where the trees and bushes were bare (leafless) and the ground cover was completely dried out.

Our study aimed to investigate the Nepticulidae in the tropical dry forests and address the question of what these abundant minuscule grey moths were during the dry season along the Pacific coast of Honduras. To accomplish this, it was necessary to dissect the specimens collected in the tropical dry forests, identify the taxa, and describe new species. 

The study advances our understanding of Nepticulidae taxonomy and morphology, while also enhancing our knowledge of the biodiversity within the tropical dry forests in Central America and the Neotropics. Furthermore, it reveals a previously unknown phenomenon specific to these dry forests.

## 2. Materials and Methods

**Materials.** The materials for this paper were obtained by one of the authors’ team. Since 2023, Prof. Dr. Jonas R. Stonis, Senior Researcher at NRC, has been visiting the Delegation of the European Union to Honduras and conducting voluntary research on the biological diversity of Honduran forests. During this mission, he sampled leaf-mining Lepidoptera. This initiative was part of two long-term programs between the European Union and Honduras: the Memorandum of Understanding between the Republic of Honduras and the European Union (“Forest Partnership”) and the Multiannual Indicative Program of the European Union for Honduras for 2021–2024, which includes Priority Area 1: “Sustainable Management of Natural Resources and Climate Change”, with the participation of the Honduran Institute of Forest Conservation, Protected Areas, and Wildlife (ICF). The material used in this paper will be deposited in the collection of the Museum für Naturkunde (MfN), Berlin, Germany, following publication.

**Figure 1:**
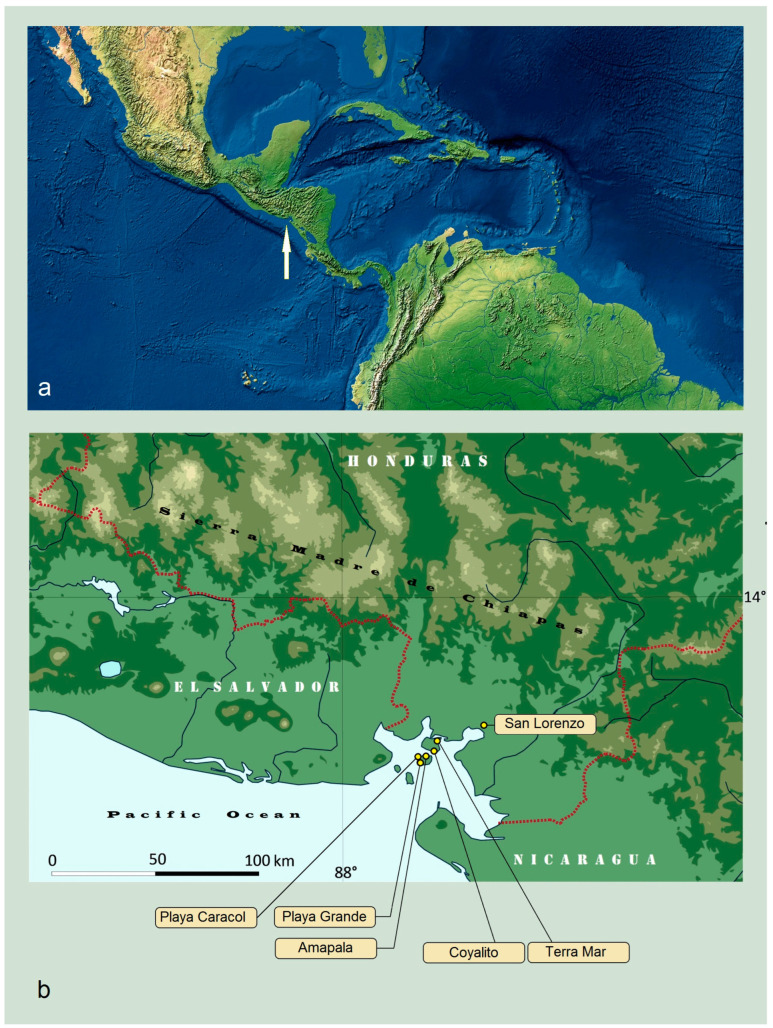
Study area: (**a**) general map (courtesy of T. Patterson, USA); (**b**) sampling locations in the tropical dry forests (map base courtesy of Virginijus Gerulaitis, Vytautas Magnus University, Lithuania).

**Figure 2:**
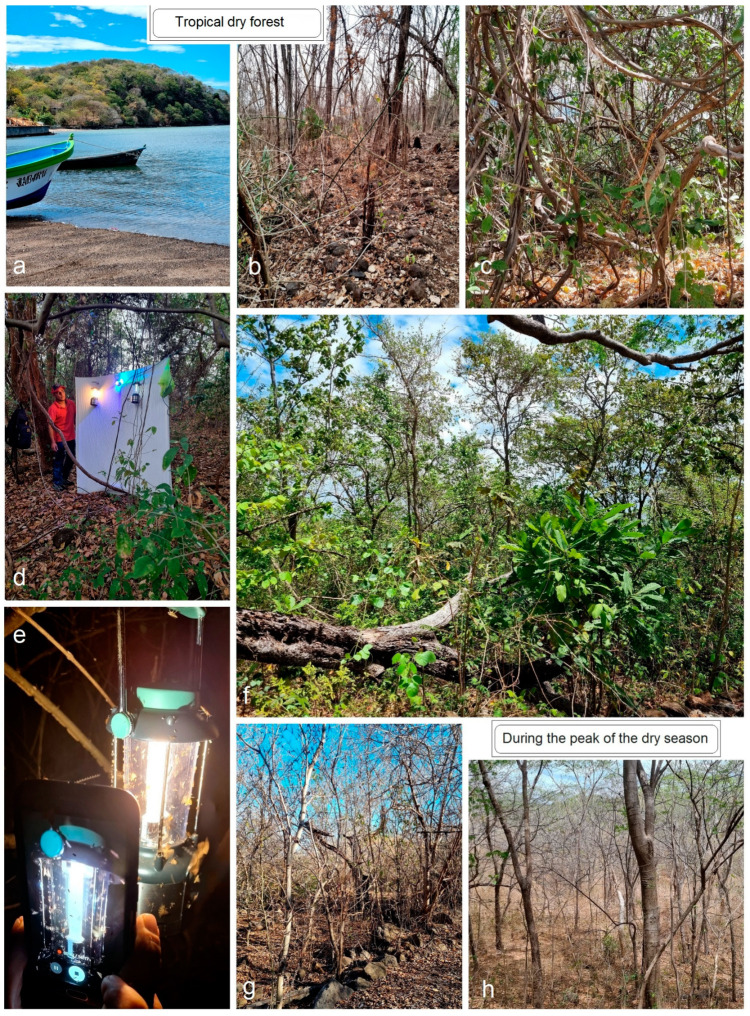
Sampling habitats in the tropical dry forests (=the Central American dry forests) during the dry season: (**a**–**c**) Isla del Tigre, Amapala, Playa Caracol, approx. 20 m; (**d**,**e**) night sampling in Isla Zacate Grande, El Moray (Terra Mar), 20 m; (**f**) Isla Zacate Grande, Coyalito (Las Piletas), ca.50 m; (**g**,**h**) same, during the peak of the dry season.

**Collecting Methods**: During our fieldwork in Honduras (Figure 1 and Figure 2), we collected micromoth using a Philips ML 220–230 V, 160 W bulb, hung in front of a white screen and powered by the electric mains. In areas without access to electric mains, we used a modern LepiLED lamp and fluorescent lanterns powered by D dry cell batteries (Figure 2d,e). The LepiLED lamp, specifically designed for collecting nocturnal moths, is lightweight, compact, and operates on 5–13 V DC voltage from power bank batteries [31].

**Specimen Dissection and Documentation.** The methods and protocols for specimen dissections, species identification, and description followed the procedures detailed in earlier publications [7,8,9]. During dissection, male genital capsules were extracted following abdomen maceration in 10% KOH, subsequent cleaning, and mounting with the ventral side facing up. In many cases, the phallus was dissected and mounted alongside the genital capsule. Abdominal pelts were not consistently preserved in this study. Permanent preparations on microscope slides were photographed and examined using a Leica DM2500 microscope equipped with a Leica DFC420 digital camera. Adult specimens were measured and examined using a Lomo stereoscopic microscope MBS-10, with images captured using a Leica S6D stereoscopic microscope paired with a Leica DFC290 digital camera. The illumination of adult specimens was achieved using a stereomicroscope ring light LED 60, directly attached to the stereo microscope lens, with adjustable illumination intensity and a color temperature range of 7000 to 11,000 K, providing 8000 Lux illumination at a 100 mm distance. Images of *Acalyptris podenasi* sp. nov. were captured using a Canon EOS R5 digital camera with a Canon MP-E 65 mm macro lens and Mitutoyo M Plan Apo 10× and 20× lenses, stacked using Zerene Stacker (PMax algorithm).

**Molecular Analysis.** The preparation of fragments of the mitochondrial DNA cytochrome c oxidase subunit 1 (mtDNA CO1-5′) for sequencing is described in Orlovskytė et al. [32]. The automated Sanger sequencing was performed in BaseClear B.V. (Leiden, The Netherlands) with the ABI 3730 xl 96-capillary DNA analyzer (Applied Biosystems, Foster City, CA, USA). The 606–657-base-pair (bp)-long CO1-5′ sequences, aligned using BioEdit v.7.2.5 [33], were deposited in the NCBI GenBank database [34] (the accession IDs: PQ008650–PQ008657) and the public BOLD platform [35] (the process IDs: ACAL001-24–ACAL008-24). The molecular data of other Nearctic and Neotropical *Acalyptris* Meyrick species were available from our previous study [20], while the sequences of the Palaearctic species and the Central American *A. janzeni* van Nieukerken & Nishida were accessible from BOLD. MEGA v.7 software [36] was used to evaluate pairwise distances and to construct the Neighbor-Joining (NJ) (10,000 bootstrap replicates, TN93 + G + I model) and the Maximum Likelihood (ML) (10,000 bootstrap replicates, GTR + G + I model) trees. The Bayesian phylogenetic analysis was performed with the GTR + G + I model and run for 5–10 million generations with the MrBayes v.3.2.3 program [37]. The Bayesian trees were processed with FigTree v.1.4.4 [38].

**Abbreviation for Institutions and Specimen Depositories**. BRG—Biosystematics Research Group, currently based at the State Research Institute Nature Research Centre (NRC), Vilnius, Lithuania; MfN—Museum für Naturkunde, formerly known as the Museum für Naturkunde der Humboldt Universität zu Berlin or Museum für Naturkunde/Leibniz-Institut für Evolutions und Biodiversitätsforschung, Berlin, Germany; NRC—the State Research Institute Nature Research Centre, Vilnius, Lithuania; ZMUC—Zoological Museum, Natural History Museum of Denmark, Copenhagen, Denmark.

## 3. Results

### 3.1. Taxonomic Documentation of Acalyptris Meyrick Species from the Tropical Dry Forests of the Pacific Coast of Honduras

**Figure 3:**
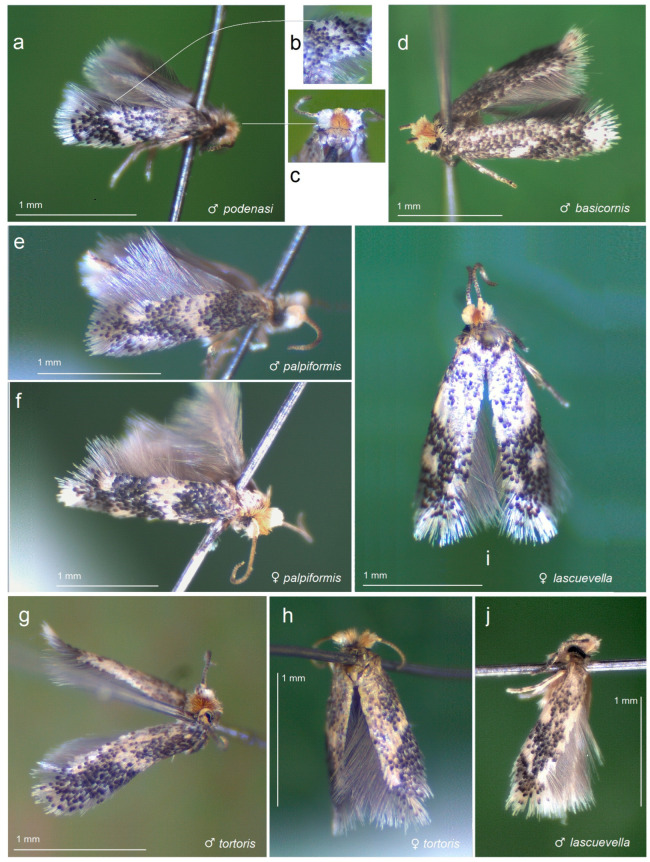
Adults of *Acalyptris* spp. discovered in the tropical dry forests: (**a**–**c**) *A. podenasi* Stonis, Dobrynina & Remeikis, sp. nov.; (**d**) *A. basicornis* Remeikis & Stonis; (**e**,**f**) *A. palpiformis* Stonis, Remeikis & Diškus, sp. nov.; (**g**,**h**) *A. tortoris* Stonis, Diškus & Dobrynina, sp. nov.; (**i**,**j**) *A. lascuevella* Puplesis & Robinson.

Among the materials obtained by us in the dry season from the tropical dry forests of Honduras, minuscule, grey-colored moths were found to be abundant and distinctly predominated (Figure 3). Laboratory dissections of the genitalia structures showed that all examined species possess the complex of morphological characters characteristic of the genus *Acalyptris* Meyrick [39], including the unique lateral apodemes. The wing venation of the examined species was neither studied nor deemed necessary, as the genitalia structures clearly exhibited the differences. Additionally, wing venation provides minimal diagnostic value due to its highly reduced state and resulting uniformity. 

Our morphological studies led to the identification of five species. Two species, *Acalyptris lascuevella* Puplesis & Robinson and *A. basicornis* Remeikis & Stonis, were new to the Honduran fauna but previously known from neighboring countries: *A. lascuevella* from Belize [12] and Mexico [9,15], and *A. basicornis* from Guatemala [9,13]. Based on the specimens sampled in Honduras and subsequently examined during the preparation of this paper, we also described three new species: *Acalyptris podenasi* sp. n., *A. palpiformis* sp. n., and *A. tortoris* sp. n. Along with our morphological examination, we have barcoded all new species, as well as *A. lascuevella*, which was exceptionally abundant in the available materials and exhibited some morphological variation.

***Acalyptris podenasi* Stonis, Dobrynina & Remeikis, sp. nov.**

https://zoobank.org/NomenclaturalActs/535d8ed4-2caf-4ef2-a959-c3e29c30c260

(accessed on 24 August 2024)

**Figure 4:**
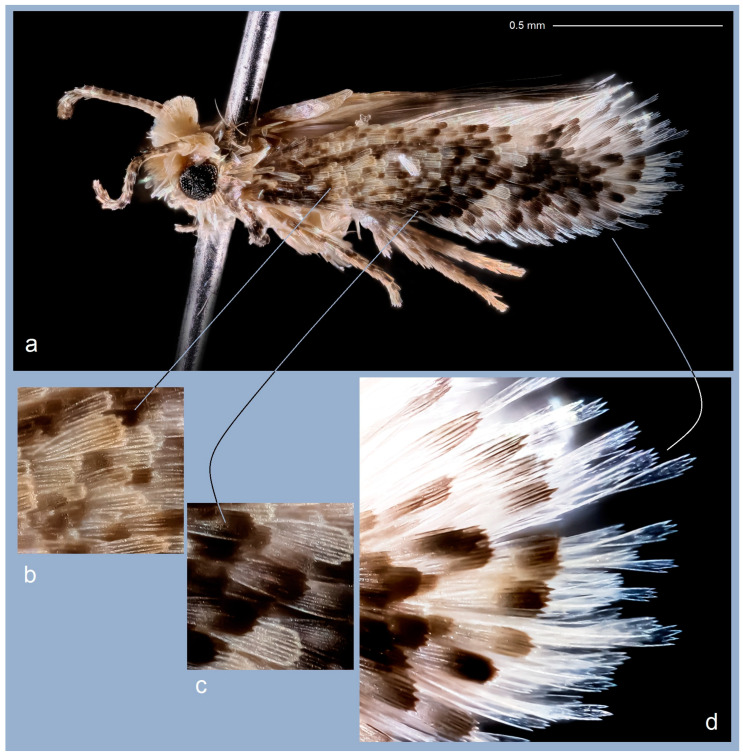
Adult of *Acalyptris podenasi* Stonis, Dobrynina & Remeikis, sp. nov.: (**a**) general view; (**b**,**c**) scales of the forewing; (**d**) fringe (courtesy of Sigitas Podėnas, NRC).

**Diagnosis.** The exact taxonomic position of this new species within the *Acalyptris* species groups is currently not fully understood (see Discussion). Tentatively, it is placed in the *A. murex* species group as a satellite species. Externally, *A. podenasi* sp. nov. (Figure 3a–c and Figure 4) closely resembles the very similar *A. lascuevella* Puplesis & Robinson, 2000 (see Figure 3i,j), making them easily confused based solely on external characters. In male genitalia, *A. podenasi* sp. nov. can be distinguished from all other *Acalyptris* species, including *A. lascuevella*, by the combination of a short but wide genital capsule, a slender transtilla with small sublateral processes, a broadly rounded pseuduncus caudally, an anchor-shaped juxta, and a phallus with one distally pointed carina and one rounded or truncate apical lobe. Notably, the anchor-shaped juxta alone can provide the immediate identification of *A. podenasi* sp. nov. Among the female genitalia features, *A. podenasi* sp. nov. is characterized by an irregular vaginal sclerite, a pair of pointed vaginal lobes, and unique signa with unusually large cells.

**Barcodes.** We barcoded four male paratype specimens from the Pacific coast of Honduras, 11–16 February 2023 and 18–19 March 2023 (with genitalia slides DV158, DV162, DV183, and RA1148); sequences are available in GenBank (the accession IDs: PQ008650–PQ008653) and BOLD (the process IDs: ACAL001-24–ACAL004-24).

**Figure 5:**
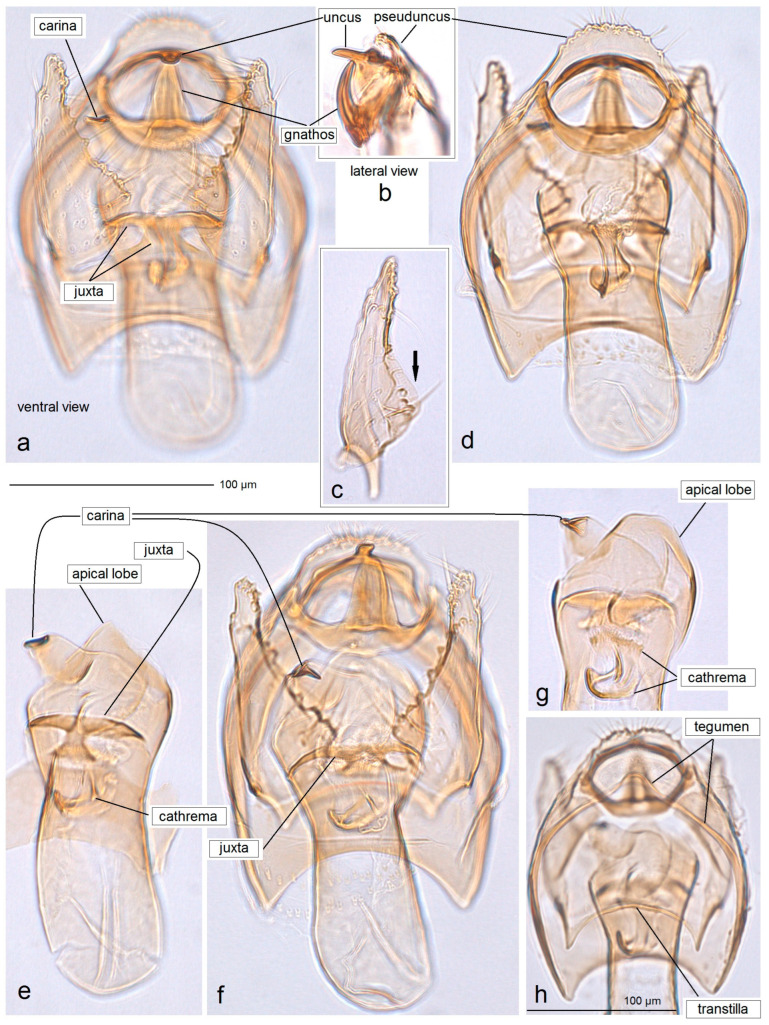
Male genitalia of *Acalyptris podenasi* Stonis, Dobrynina & Remeikis, sp. nov.: (**a,d**) genitalia slide no. DV151, holotype; (**b,c**,**g**) genitalia slide no. DV154, paratype; (**e**) genitalia slide no. DV169, paratype; (**f**,**h**) genitalia slide no. DV177 (MfN).

**Male** (Figure 4). The forewing length ranges from 1.3 to 1.5 mm, with a wingspan of 3.0 to 3.3 mm (n = 28). *The head*: The palpi are silvery cream; the frontal tuft varies from brownish-orange to pale orange; the collar consists of two somewhat indistinct tufts of piliform scales; the scape is unusually large, cream-colored with sparse scattered scales; the antenna is shorter than one-third of the length of the forewing; the flagellum is blackish-grey to pale grey, silvery-cream on the underside. *The thorax*: The tegula is silvery-cream, speckled with dark scales; the thorax is predominantly silvery-cream with a few dark scales. The forewing is densely irrorated with black-brown scales; it bears two large, irregular, almost merging silvery-cream or silvery-white (occasionally cream) postmedian costal and dorsal spots, or a silvery-cream to silvery-white oblique postmedian fascia; sometimes the dark scales of the forewing are less developed in the basal half, giving it a silvery-cream appearance; the fringe is cream to silvery-cream or silvery-white, pale grey on the tornus, with a distinctive fringe line; the underside of the forewing is blackish-grey. The hindwing and its fringe are greyish-cream to cream; the apical third is covered with tiny pale grey scales (androconia), visible at certain angles. The legs are glossy cream, covered with glossy grey scales on the upper side. *The genitalia* (Figure 5): The capsule measures 180–190 µm in length and 140–175 µm in width. The pseuduncus is widely rounded. The uncus is inverted Y-shaped, with a short, ventrally directed process. The gnathos possesses a stout caudal process, a slender median plate, and well-developed lateral arms. The lateral apodeme is relatively short and slender, with an enlarged, lobe-like caudal part. The valva is 105–110 µm long, almost triangular, heavily papillated medially, with a subbasal bulge. The transtilla features a slender transverse bar and tiny, pointed sublateral processes. The vinculum is relatively wide but short, with two short, triangular lateral lobes. The phallus is 175–190 µm long and 60–75 µm wide, with a rounded or truncated apical lobe and an irregularly shaped, distally thickened carina; cornuti are absent. The juxta is distinctive and thickened, anchor-shaped (note: the shape may distort during inaccurate dissections).

**Figure 6:**
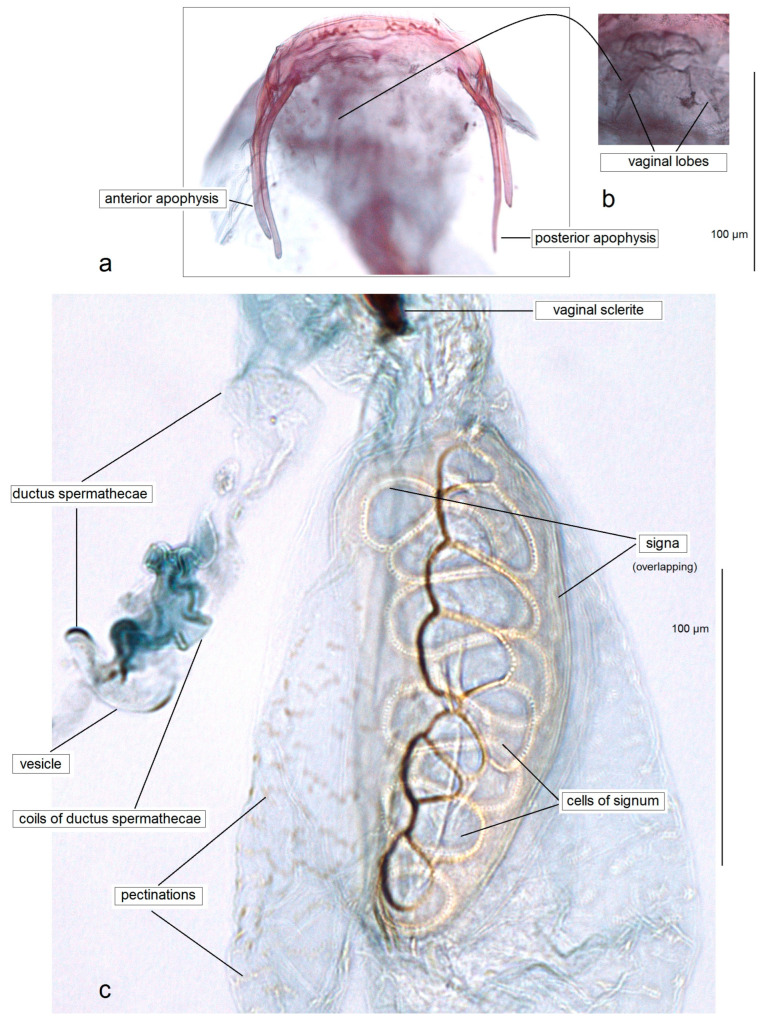
Female genitalia of *Acalyptris podenasi* Stonis, Dobrynina & Remeikis, sp. nov.: (**a**,**b**) genitalia slide no. RA1153, paratype; (**c**) genitalia slide no. DV187, paratype (MfN).

**Figure 7:**
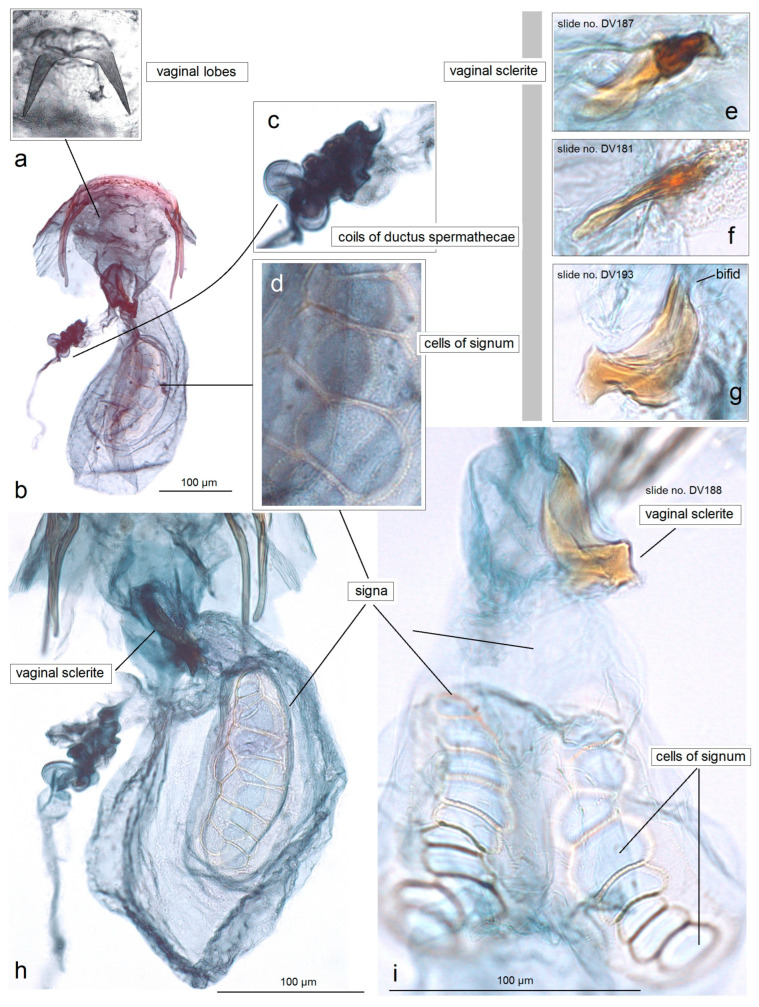
Female genitalia of *Acalyptris podenasi* Stonis, Dobrynina & Remeikis, sp. nov.: (**a**–**d**) genitalia slide no. RA1153, paratype; (**e**–**g**) slide nos DV181, DV187, DV193, paratypes, vaginal sclerites; (**h**) genitalia slide no. RA1154, paratype; (**i**) genitalia slide no. DV188, paratype (MfN).

**Female.** Externally, the female is similar to the male. Genitalia (Figure 6 and Figure 7): The total length of the genitalia is approximately 440 µm. The abdominal apex is wide and slightly rounded caudally, with two rows of short setae. Both the anterior and posterior apophyses are slender, with the posterior ones distinctly longer. The vestibulum contains an irregular vaginal sclerite. The corpus bursae is oval-shaped, densely covered with minute pectinations; the signa have exceptionally large cells, up to 35–40 µm in width. The ductus spermathecae is with compressed, heavily thickened, irregular coils and a lobe-shaped, rounded vesicle.

**Bionomics.** The host plant remains unknown. Adults are attracted to light. All currently available specimens were abundantly collected at a light trap from early February to the end of March, during the peak of the dry season. Otherwise, the biology of this species is unknown.

**Distribution.** This new species occurs and appears to be very common in the tropical dry forests along the Pacific coast of Honduras, including the islands of the Gulf of Fonseca.

**Etymology.** The species is named after our colleague Prof. Dr. Sigitas Podėnas, an entomologist specializing in Tipulimorpha, the Director of the State Research Institute Nature Research Centre (NRC), and an Academician of the Lithuanian Academy of Sciences, in recognition of his passion for studying insect diversity. We also greatly appreciate his efforts in providing the wonderful image of the nepticulid moth included in this paper (see Figure 4).

**Type material.** Holotype: ♂, HONDURAS, The Pacific, Isla Zacate Grande, Coyalito (Las Piletas), ca.50 m, 13°18′45.0′′ N, 87°36′59.3′′ W, 23–24 March 2023, leg. J.R. Stonis, genitalia slide no. DV151♂ (MfN). Paratypes (85 ♂, 18 ♀): 2 ♀, same label data as holotype, genitalia slide no. RA1154♀ (MfN); 22 ♂, 5 ♀, Isla Zacate Grande, El Moray (Restaurante Terra Mar), 20 m, 13°21′28.5′′ N, 87°36′06.5′′ W, 15–16 February 2023, leg. J.R. Stonis, genitalia slide nos DV154♂, DV163♂, DV164♂, DV173♂, DV182♂, DV183♂ (only genitalia, no adult preserved*), DV184♂, DV187♀, DV192♀, DV193♀, RA1148♂ (only genitalia, no adult preserved*), RA1165♂, RA1168♂, RA1172♂, RA1174♂ (MfN); 23 ♂, 3 ♀, El Moray (Restaurante Terra Mar), ca.10 m, 13°21′33.6′′ N, 87°35′57.5′′ W, 18–19 March 2023, leg. J.R. Stonis, genitalia slide nos DV162♂ (only genitalia, no adult preserved*), DV179♂, DV181♀, DV186♂, DV189♂, RA1153♀, RA1166♂, RA1167♂, RA1173♂ (MfN); 28 ♂, 4 ♀, Isla Zacate Grande, El Moray (near Terra Mar), 25 m, 13°21′28.5′′ N, 87°36′06.5′′ W, 11–12.ii.2023, leg. J.R. Stonis, genitalia slide nos DV150♂, DV152♂, DV154♂, DV158♂ (only genitalia, no adult preserved*), DV161♂, DV166♂, DV167♂, DV168♂, DV175♂, DV178♂, DV180♂, RA1169♂, RA1170♂ (MfN); 9 ♂, 2 ♀, El Moray (near Restaurante Terra Mar), ca.20 m, 13°21′30.1′′ N, 87°36′05.6′′ W, 20 March 2023, leg. J.R. Stonis, genitalia slide nos DV177♂, DV190♂, DV194♂, RA1164♂, RA1171♂, (MfN); 2 ♂, 3 ♀, the Pacific, San Lorenzo, 1.5 km E by Pan American Hwy (left side), approx. 40 m, 13°25′59.2′′ N, 87°25′24.7′′ W, 6–7 February 2023, leg. J.R. Stonis, genitalia slide nos DV169♂, DV172♂, DV188♀ (MfN) (* taken for DNA studies).

**Remarks.** Identifying species from the tropical dry forests of Honduras based solely on external characteristics can be challenging or even impossible. A sizeable additional collection was excluded from the type series and deposited at the BRG/NRC. The material, left undissected, comprises male and female specimens of two externally similar species, *Acalyptris podenasi* sp. nov. and *A. lascuevella* Puplesis & Robinson.

***Acalyptris palpiformis* Stonis, Remeikis & Diškus, sp. nov.**

https://zoobank.org/NomenclaturalActs/FEDC4E67-DE3B-4DC6-995E-31D58D3F2D39

(accessed on 24 August 2024)

**Diagnosis.** *Acalyptris palpiformis* sp. nov. is assigned to the *A. trifidus* species group (see Molecular Considerations). Externally, this new species is characterized by two pale postmedian spots and a pale antemedian fascia, setting it slightly apart from other grey-speckled *Acalyptris* species. In the male genitalia, *A. palpiformis* sp. nov. can be distinguished from all other *Acalyptris* species, including those of the *A. trifidus* group, by the presence of two palpi-like lateral processes of the uncus, as well as a slender valva, transtilla with long pointed sublateral processes, and unique long carinae of the phallus. In the female genitalia, *A. palpiformis* sp. nov. is characterized by unusually long posterior apophyses, large specific vaginal lobes, and laterally thickened vaginal sclerite.

**Barcode.** We barcoded one male paratype specimen from Terra Mar, Isla Zacate Grande, Honduras, 20 March 2023 (with genitalia slide no. RA1151); the sequence is available in GenBank (the accession ID: PQ008656) and BOLD (the process ID: ACAL007-24).

**Figure 8:**
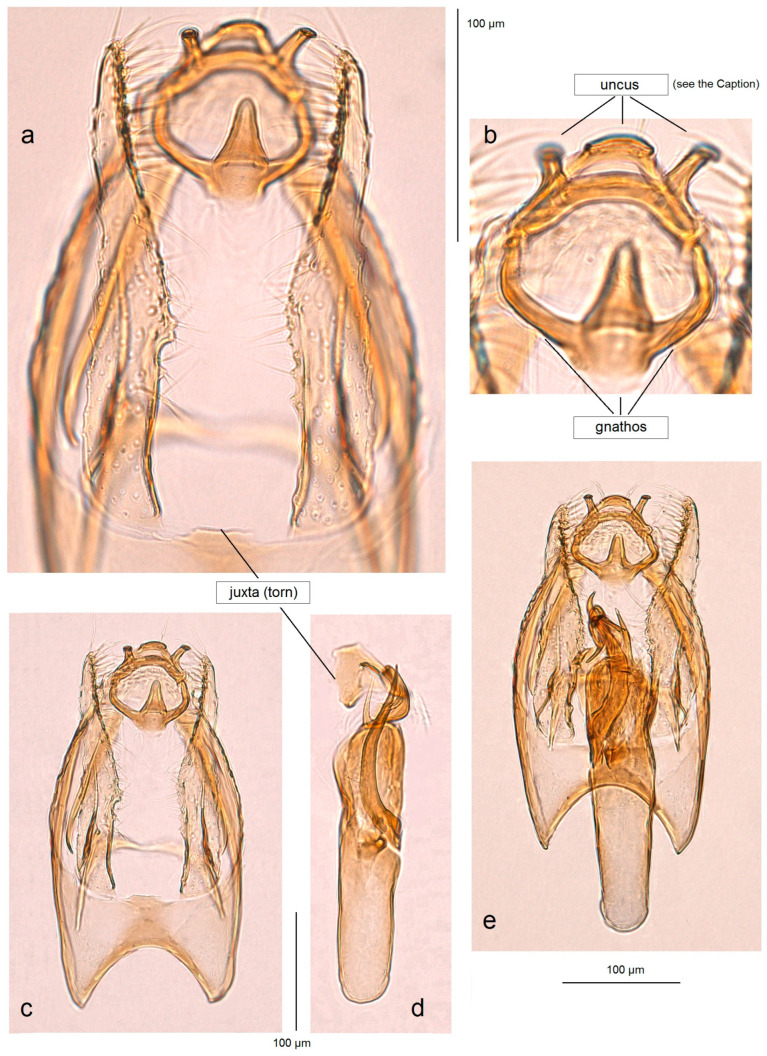
Male genitalia of *Acalyptris palpiformis* Stonis, Remeikis & Diškus, sp. nov.; (**a**–**d**) genitalia slide no. RA1158, paratype; (**e**) genitalia slide no. RA1156, holotype (MfN).

**Figure 9:**
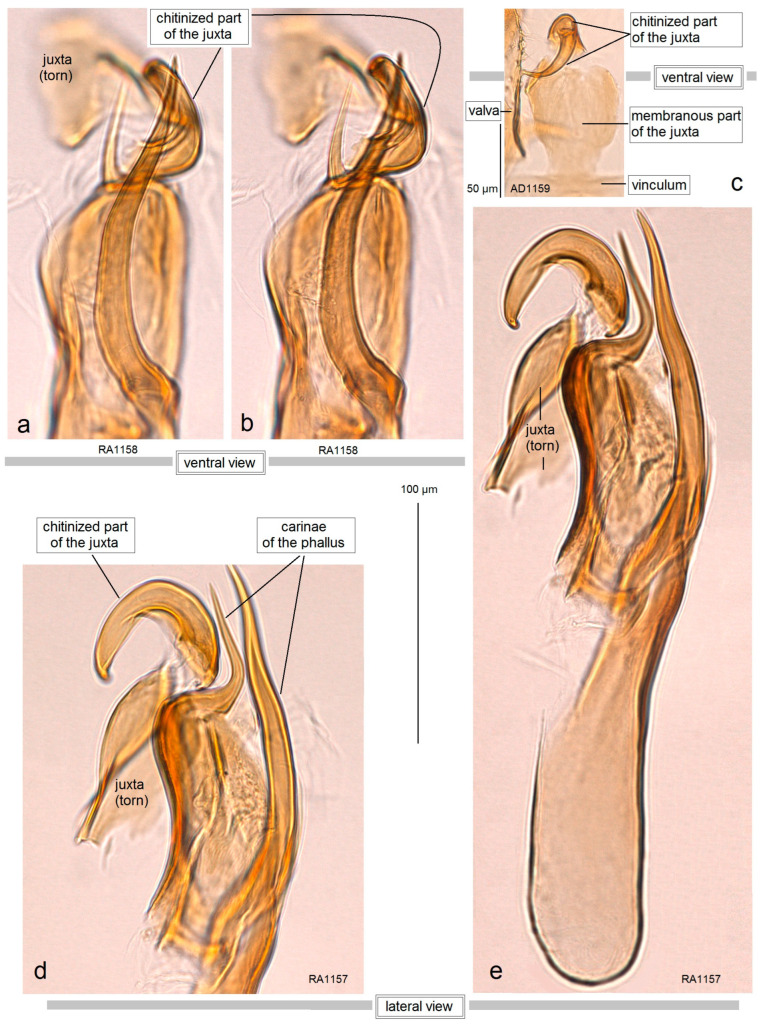
Phallus and juxta of *Acalyptris palpiformis* Stonis, Remeikis & Diškus, sp. nov.; (**a**,**b**) genitalia slide no. RA1158, paratype; (**c**) genitalia slide AD1159, paratype; (**d**,**e**) genitalia slide no. RA1157, paratype (MfN).

**Male** (Figure 3e). The forewing length ranges from 1.5 to 1.8 mm; wingspan from 3.5 to 4.0 mm (n = 8). *Head*: Palpi cream; frontal tuft pale beige-orange; collar comprised of two tufts of ochre-cream piliform scales; scape yellowish-cream; antenna longer than one third the length of the forewing; upper side of flagellum dark grey with purple iridescence, pale yellowish-grey underside. *Thorax*: Tegula cream, speckled with dark brown-grey scales; thorax yellowish-silvery cream, with a few brownish-grey scales. The forewing is densely irrorated with dark blackish-brown scales, with two large cream or yellowish-cream postmedian costal and dorsal spots, and cream or yellowish-cream postmedian fascia, twice wider at the dorsal margin than on the costal margin; the fringe is yellowish-cream, grey-cream to grey on the tornus; the fringe line is indistinctive or absent; the forewing underside is densely irrorated with grey-black or blackish-brown scales. The hindwing is pale grey with pale grey fringe. Legs are cream, covered with grey scales on the upper side. *Abdomen:* The upper side is glossy grey-black to black, and the underside is ochre-cream; the genital plates are ochre-cream; the anal tuft inconspicuous, cream. *Genitalia* (Figure 8 and Figure 9): The capsule is 310–320 µm long, 170–175 µm wide. The pseuduncus is triangular, widely rounded distally. The uncus is complex, consisting of a main body with palp-like lateral processes and a median lobe attached to the main body by lateral arms. The gnathos possesses a stout caudal process, small angular median plate, and well-developed lateral arms. The lateral apodeme is long, rod-like. The valva is 195–210 µm long, slender and slightly sinuous. The transtilla has relatively long, pointed sublateral processes and a transverse bar. The vinculum is relatively short, with two large triangular lateral lobes. The phallus is 290–325 µm long, with two large horn-like pointed carinae: one smaller and curved, another very long and slightly sinuous, almost straight; cornuti absent. The juxta is attached to the phallus, with a curved hook-like distal part and proximal plate.

**Figure 10:**
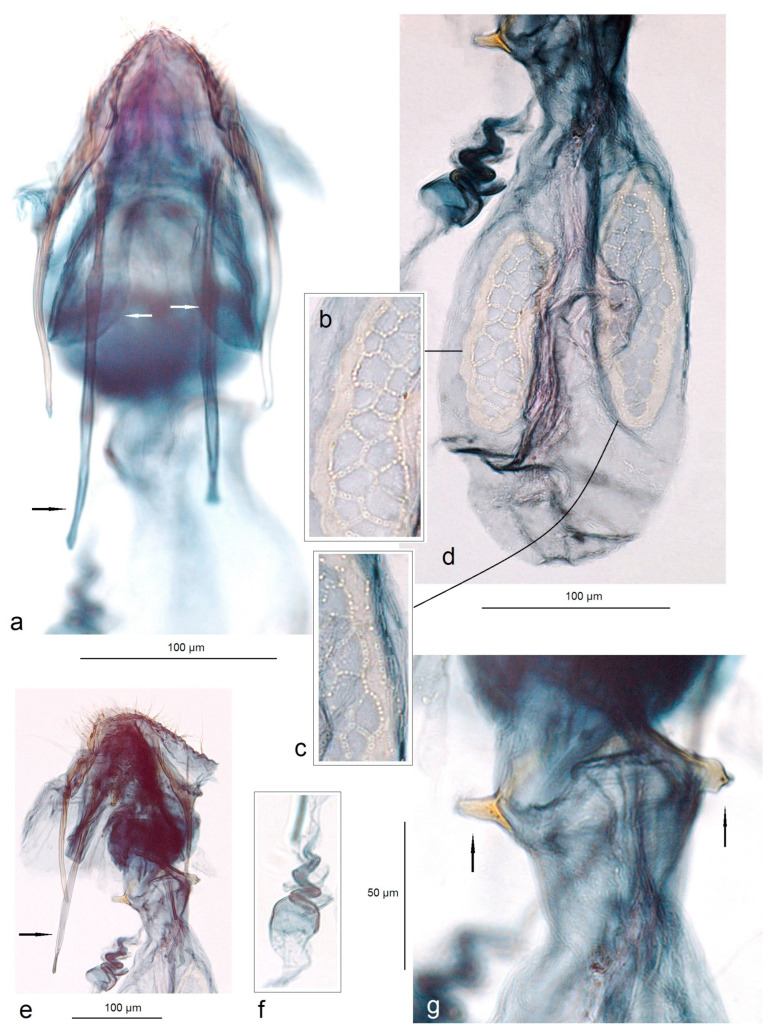
Female genitalia of *Acalyptris palpiformis* Stonis, Remeikis & Diškus, sp. nov.; (**a**) genitalia slide no. RA1161, paratype; (**b**–**d**) genitalia slide no. RA1155, paratype; (**e**–**g**) genitalia slide no. RA1155, paratype (MfN).

**Female** (Figure 3f)**.** Externally, similar to the male. The forewing length is 1.5–1.8 mm; the wingspan is 3.5–4.0 mm (n = 4). The antenna is significantly shorter than one third the length of the forewing. *Genitalia* (Figure 10): The total length of the genitalia is ca. 470 µm. The abdominal apex is triangular, caudally rounded or almost truncated. Both the anterior and posterior apophyses are slender, but the posterior ones are distinctly longer. The vestibulum is with an irregular, laterally chitinized vaginal sclerite and large vaginal lobes. The corpus bursae is oval-shaped, without or with little pectinations; the signa is with large cells. The ductus spermathecae is 3–4 heavily thickened coils and lobe-shaped.

**Bionomics.** The host plant is unknown. Adults are attracted to light. All currently available specimens were collected at a light trap from February to March, during the peak of the dry season. Otherwise, the biology of this species remains unknown.

**Distribution.** Currently, this new species is known only from tropical dry forests along the Pacific coast of Honduras, including the islands of the Gulf of Fonseca.

**Etymology.** The species name is derived from the Latin word “palpus” (meaning a palp), referring to the male genitalia characterized by the unique uncus with two palp-like processes.

**Material examined.** Holotype: ♂, HONDURAS, The Pacific, Isla del Tigre, Amapala, Playa Grande, approx. 40 m, 13°16′32.3′′ N 87°39′37.5′′ W, 13–14 March 2023, leg. J.R. Stonis, genitalia slide no. RA1156♂ (MfN). Paratypes (7 ♂, 2 ♀): 1 ♀, Isla del Tigre, Amapala, Playa Caracol, approx. 20 m, 13°16′41,1′′ N, 87°39′29.5′′ W, 18 February 2023, leg. J.R. Stonis, genitalia slide no. RA1161♀ (MfN); 1 ♂, 1 ♀, Isla del Tigre, Amapala, approx. 10 m, 13°17′41.3′′ N, 87°38′40.6′′ W, 16–17 March 2023, genitalia slide no. RA1155♀, leg. J.R. Stonis (MfN); 2 ♂, Isla Zacate Grande, Coyalito (Las Piletas), ca.50 m, 13°18′45.0′′ N, 87°36′59.3′′ W, 23–24 March 2023, leg. J.R. Stonis, genitalia slide nos DV155♂, AD1159♂ (MfN); 3 ♂, Isla Zacate Grande, El Moray (Restaurante Terra Mar), 20 m, 13°21′28.5′′ N, 87°36′06.5′′ W, 15–16 February 2023, leg. J.R. Stonis, genitalia slide nos RA1157♂, RA1158♂ (MfN); 1 ♂, Isla Zacate Grande, El Moray (near Rest. Terra Mar), ca.20 m, 13°21′30.1′′ N, 87°36′05.6′′ W, 20 March 2023, leg. J.R. Stonis, genitalia slide no. RA1151♂ (MfN).

***Acalyptris tortoris* Stonis, Diškus & Dobrynina, sp. nov.**

https://zoobank.org/NomenclaturalActs/C1B4FEB0-E2C1-4172-8275-61724A1B5907

(accessed on 24 August 2024)

**Figure 11:**
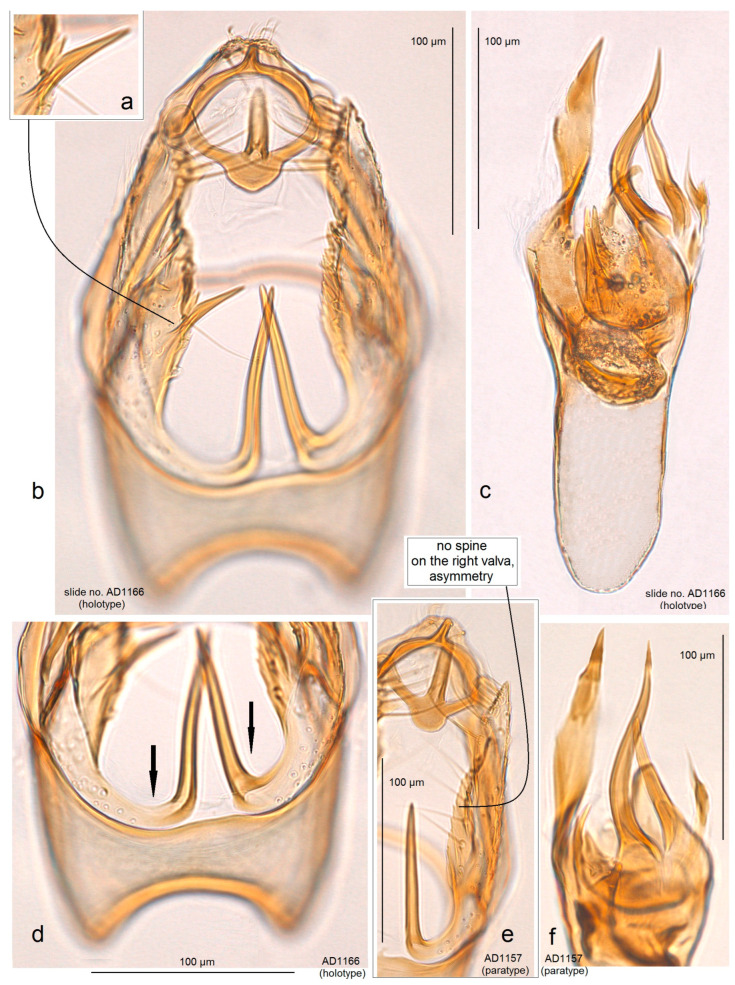
Male genitalia of *Acalyptris tortoris* Stonis, Diškus & Dobrynina, sp. nov.; (**a**–**d**) genitalia slide no. AD1188, holotype; (**e**,**f**) genitalia slide no. AD1157, paratype (MfN).

**Diagnosis.** *Acalyptris tortoris* sp. nov. is assigned to the *A. fortis* species group (see Discussion). Externally, this new species is distinguished by an irregular and indistinct oblique cream median fascia and a cream or beige-cream dorsal margin to the forewing, traits that resemble those of *Acalyptris lascuevella* Puplesis & Robinson. In the male genitalia, *A. tortoris* sp. nov. is easily distinguished from all other *Acalyptris* species, including those of the *A. fortis* group, by the valva with unique processes (Figure 11a,b). In the female genitalia, *A. tortoris* sp. nov. is characterized by a unique comb-like vaginal sclerite and a rounded ventral plate of the ovipositor.

**Barcode.** We barcoded one male paratype specimen from Playa Caracol, Honduras, 18.ii.2023 (with genitalia slide DV153); the sequence is available in GenBank (the accession ID: PQ008657) and BOLD (the process ID: ACAL008-24).

**Figure 12:**
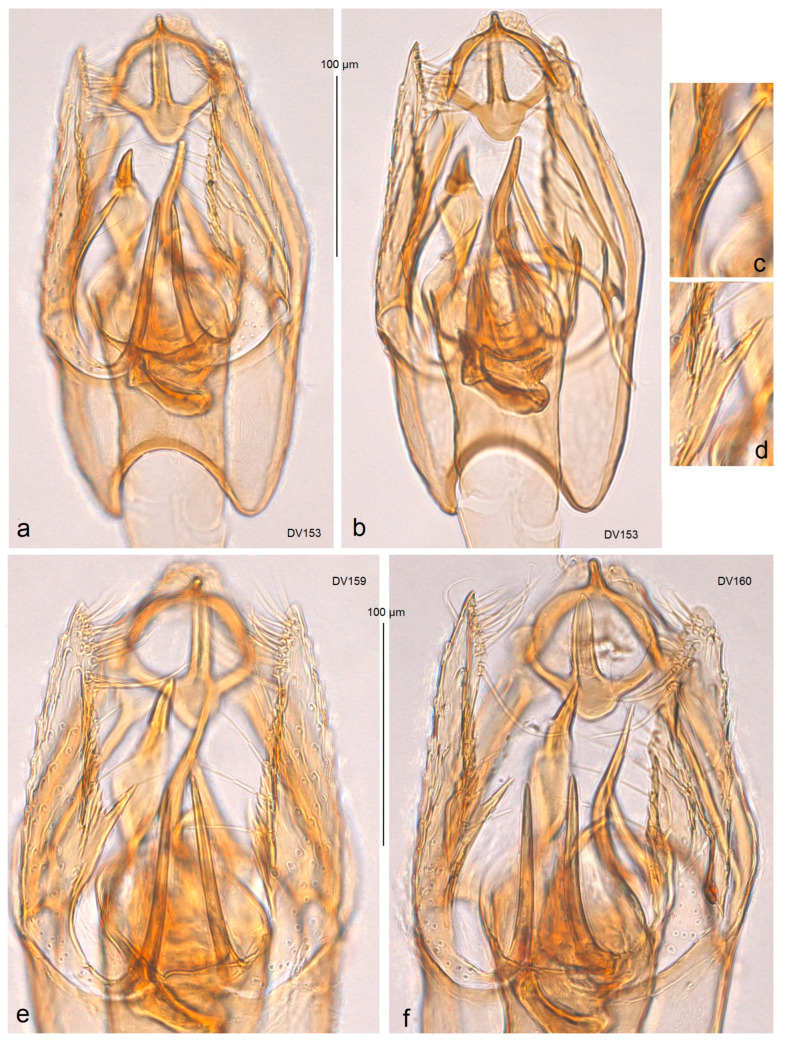
Male genitalia of *Acalyptris tortoris* Stonis, Diškus & Dobrynina, sp. nov.; (**a**,**b**) genitalia slide no. DV153, paratype; (**c**–**e**) genitalia slide no. DV159, paratype; (**f**) genitalia slide no. DV160 (MfN).

**Male** (Figure 3g). The forewing length is 1.3–1.5 mm; the wingspan is 3.0–3.5 mm (n = 3). *Head*: The palpi cream; the frontal tuft is pale beige-orange; the collar is comprised of two tufts of ochre-cream piliform scales; the scape is cream; the antenna is significantly shorter than one third the length of the forewing; the flagellum is grey on the upper side, but cream on the underside. *Thorax*: The tegula is cream, speckled with dark brown-grey scales; the thorax is yellowish-cream with a few brownish-grey scales. The forewing is densely irrorated with dark, blackish-brown scales and of an irregular and indistinctive, oblique, cream median fascia; the latter can be undeveloped; the dorsal margin of the forewing is cream to yellowish cream; the fringe is cream to pale grey on the tornus; the fringe line is distinctive; the forewing underside is densely irrorated with dark brown-grey or blackish-grey scales. The hindwing and its fringe are pale grey. The legs are beige-cream, often covered with black scales on the upper side. *Genitalia* (Figure 11 and Figure 12): The genitalia capsule is ca. 280 µm long, 170–175 µm wide. The pseuduncus is narrowed and truncated distally. The uncus is inverted Y-shaped. The gnathos possesses a relatively long and slender process, small anteriorly rounded median plate, and well-developed lateral arms. The lateral apodeme is long and slender, rod-like, caudally wide. The valva is 175–180 µm long, with a long slender, distally pointed basal process; the left valva possesses a median spine (a short spine-like process), while the right valva has no spine-like process (a case of genital asymmetry). The transtilla is with slender, relatively long sublateral processes. The vinculum is with two short but wide lateral lobes and relatively shallow anterior excavation. The phallus is 275–280 µm long, with two large, horn-like, sinuous carinae and about five shorter, spine-like carinae. The juxta is absent.

**Figure 13:**
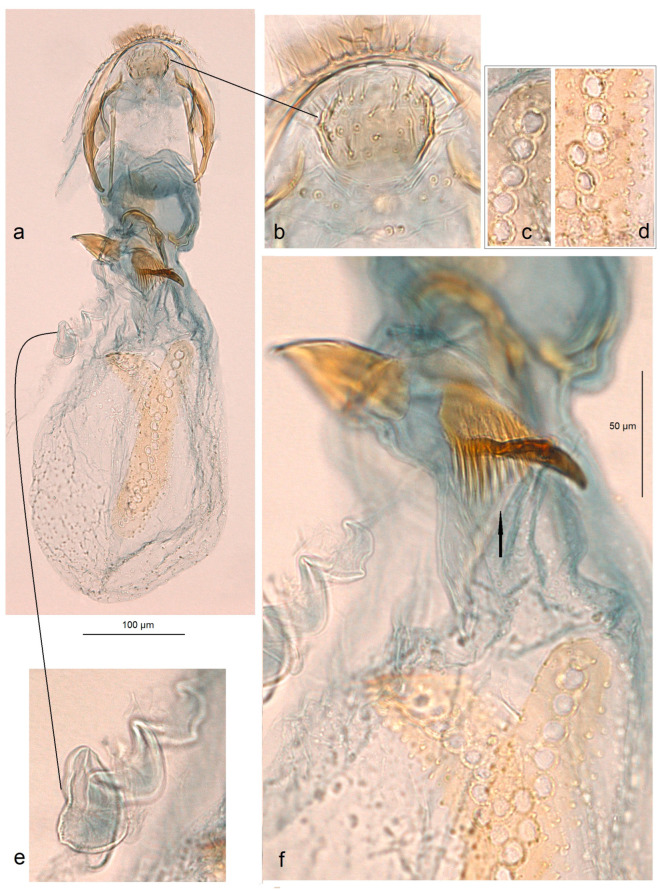
Female genitalia of *Acalyptris tortoris* Stonis, Diškus & Dobrynina, sp. nov., genitalia slide no. DV153, paratype: (**a**) general view; (**b**) thickened ventral plate on the ovipositor; (**c**,**d**) signum cells; (**e**) coils of ductus spermathecae; (**f**) vaginal sclerite (MfN).

**Female** (Figure 3h)**.** Externally similar to the male. *Genitalia* (Figure 13): The total length of the genitalia is 620 µm. The abdominal apex is widely rounded, with a rounded thickened plate in the middle ventrally. The anterior and posterior apophyses are almost equal in their length. The vestibulum is a complex, laterally chitinized vaginal sclerite; the median part of the latter is comb-like. The corpus bursae is oval-shaped, with pectinations; each signum with one row of small cells. The ductus spermathecae is 2–3 coils and a lobe-like vesicle.

**Bionomics**. The host plant is unknown. Adults are attracted to light. All currently available specimens were collected at a light trap from February to March, during the peak of the dry season. Otherwise, the biology of this species remains unknown.

**Distribution**. Currently, this new species is only known from tropical dry forests along the Pacific coast of Honduras, including the islands of the Gulf of Fonseca.

**Etymology.** The species name is derived from the Latin word “tortoris” (meaning torture), referring to the heavily armed genitalia with large spines in the male and the comb-like vaginal sclerite in the female genitalia.

**Material examined.** Holotype: ♂, HONDURAS, The Pacific, Isla Zacate Grande, El Moray (Restaurante Terra Mar), 20 m, 13°21′28.5′′ N, 87°36′06.5′′ W, 15–16 February 2023, leg. J.R. Stonis, AD1166♂ (MfN). Paratypes (5 ♂, 1♀): 1 ♂, same label as holotype, genitalia slide no. DV160♂ (MfN); 1 ♂, Isla Zacate Grande, El Moray (near Restaurante Terra Mar), ca.20 m, 13°21′30.1′′ N, 87°36′05.6′′ W, 20 March 2023, leg. J.R. Stonis, genitalia slide no. AD1163♂ (MfN); 2 ♂, Isla del Tigre, Amapala, Playa Caracol, approx. 20 m, 13°16′41.1′′ N, 87°39′29.5′′ W, 18 February 2023, leg. J.R. Stonis, genitalia slide nos DV153♂ (only genitalia, no pinned adult*), AD1157♂ (aberrant) (MfN); 1 ♀, Isla Zacate Grande, Coyalito (Las Piletas), ca. 50 m, 13°18′45.0′′ N, 87°36′59.3′′ W, 23–24 March 2023, leg. J.R. Stonis, genitalia slide no. AD1167♀ (MfN); 1 ♂, the Pacific, San Lorenzo, 1.5 km E by Pan American Hwy (left side), approx. 40 m, 13°25′59.2′′ N, 87°25′24.7′′ W, 6–7 February 2023, leg. J.R. Stonis, genitalia slide no. DV159♂ (MfN) (* the pinned moth has been taken and completely used for DNA studies).

***Acalyptris lascuevella* Puplesis & Robinson**

*Acalyptris lascuevella* Puplesis & Robinson, 2000 [12] (pp. 49,50).

**Figure 14:**
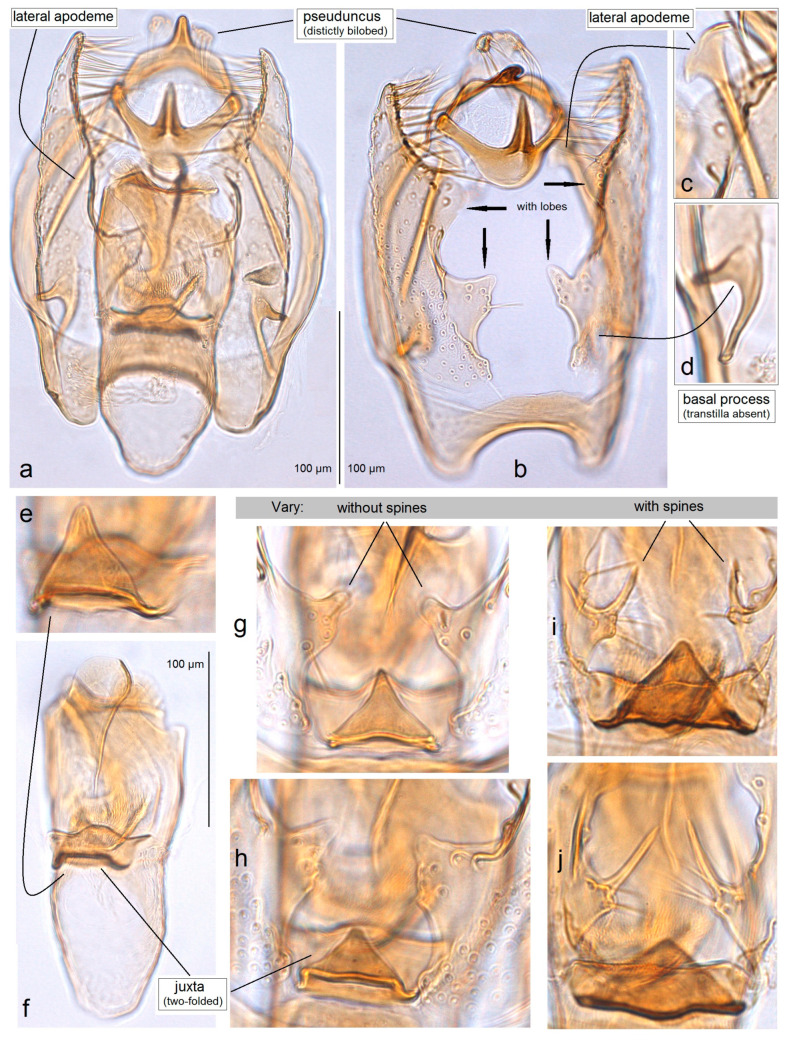
Male genitalia of *Acalyptris lascuevella* Puplesis & Robinson: (**a**) genitalia slide no. RA1152; (**b**) genitalia slide no. DV171; (**c**) genitalia slide no. DV174; (**d**) genitalia slide no. RA1152; (**e**,**f**) genitalia slide no. DV171; (**g**) genitalia slide no. DV185; (**h**) genitalia slide no. DV174; (**i**) genitalia slide no. DV176; (**j**) genitalia slide no. DV157 (MfN).

**Figure 15:**
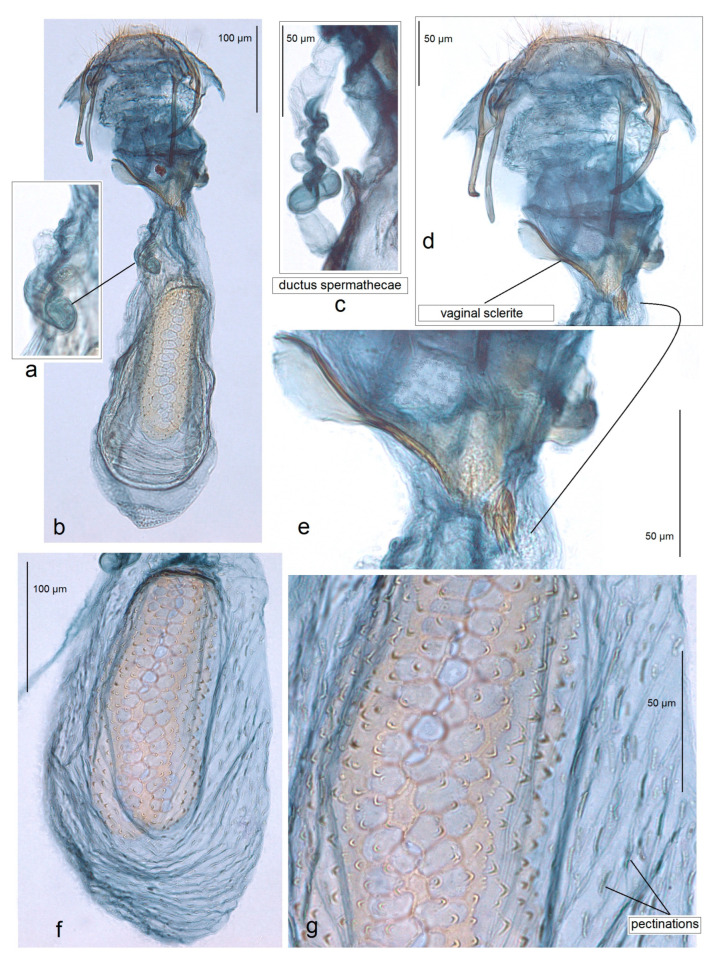
First documentation of female genitalia of *Acalyptris lascuevella* Puplesis & Robinson: (**a**,**b**) genitalia slide no. RA1162; (**c**) genitalia slide no. RA1159; (**d**,**e**) genitalia slide no. RA1162; (**f**,**g**) genitalia slide no. RA1160 (MfN).

**General data.** Host plants are unknown. The forewing length ranges from 1.5 to 1.7 mm, with a wingspan of 3.4 to 3.7 mm (n = 12♂♀) (Figure 3i,j). Originally described from the tropical moist forests of Belize [12], it was later discovered in a tropical dry forest habitat in Mexico [15]. Based on our study of large material, we report *A. lascuevella* for the first time in Honduras, marking a new distribution record and documenting the genitalia morphology (Figure 14 and Figure 15). The females of *A. lascuevella* were previously unknown; therefore, they are documented here for the first time (Figure 15).

**Barcodes.** We barcoded two male specimens from the Pacific coast of Honduras, 18 February 2023 (with genitalia slide DV170) and 14 March 2023 (with genitalia slide RA1149); the sequences are available in GenBank (the accession IDs: PQ008654, PQ008655) and BOLD (the process IDs: ACAL005-24, ACAL006-24).

**Remarks.** Our examination of *A. lascuevella* collected in the tropical dry forests of Honduras revealed variations in the morphology of the male genitalia. The basal inner lobe of the valva varied in shape and the presence of a spine; the juxta also showed slight variations among the examined specimens: in some cases, it appeared less chitinized and slender, while in others, it was strongly chitinized and distinctly triangular. It is worth noting that approximately 70–80% of the studied male genitalia of *A. lascuevella* exhibited various transitional forms. Additionally, numerous instances were observed where the right valval lobe differed in shape from the left lobe of the same specimen. Moreover, these differences did not correlate with each other or with the variations found in the molecular sequences.

For illustrations of specimens from Belize (the type locality), we recommend consulting the following publication by Puplesis and Robinson (2000) [12] (Figures 180 and 181).

**Material examined.** 13 ♂, 2 ♀, HONDURAS, The Pacific, Isla Zacate Grande, El Moray (Restaurante Terra Mar), 20 m, 13°21′28.5′′ N, 87°36′06.5′′ W, 15–16 February 2023, leg. J.R. Stonis, genitalia slide nos DV156♂, DV157♂, DV185♂, DV191♂, RA1152♂, RA1176♂, RA1179♂, RA1180♂, RA1183♂, RA1185♂, RA1186♂, RA1187♂ (MfN); 1 ♂, same locality, ca.10 m, 13°21′33.6′′ N, 87°35′57.5′′ W, 18–19 March 2023, leg. J.R. Stonis, genitalia slide no. AD1162♂ (MfN); 4♂, 1 ♀, Isla Zacate Grande, El Moray (near Terra Mar), 25 m, 13°21′28.5′′ N, 87°36′06.5′′ W, 11–12 February 2023, leg. J.R. Stonis, genitalia slide nos DV171♂, RA1177♂, RA1178♂, RA1181♂ (MfN); 1 ♂, Isla Zacate Grande, El Moray (near Rest. Terra Mar), ca.20 m, 13°21′30.1′′ N, 87°36′05.6′′ W, 20 March 2023, leg. J.R. Stonis, genitalia slide no. RA1175♂ (MfN); 3 ♂, 2 ♀, Isla del Tigre, Amapala, Playa Caracol, approx. 20 m, 13°16′41.1′′ N, 87°39′29.5′′ W, 18 February 2023, leg. J.R. Stonis, genitalia slide nos DV170♂ (only genitalia, no pinned adult*), DV174♂, RA1184♂ (MfN); 2 ♂, 1 ♀, Isla del Tigre, Amapala, Playa Grande, approx. 40 m, 13°16′32.3′′ N 87°39′37.5′′ W, 13–14 March 2023, leg. J.R. Stonis, genitalia slide nos DV176♂, RA1149♂, (MfN); 3 ♀, San Lorenzo, 1.5 km E by Pan American Hwy (left side), approx. 40 m, 13°25′59.2′′ N, 87°25′24.7′′ W, 6–7 February 2023, leg. J.R. Stonis, genitalia slide nos RA1159♀, RA1160♀, RA1162♀ (MfN); 1 ♂, Cantarranas (=San Juan de Flores), ca. 660 m, 14°15′57.0′′ N 87°01′31.4′′ W, 14°16′17.0′′ N 87°01′04.6′′ W, 18–23 April 2023, leg. J.R. Stonis, genitalia slide no. RA1182♂ (MfN) (* the pinned moth has been taken for DNA studies).

***Acalyptris basicornis* Remeikis & Stonis**

*Acalyptris basicornis* Remeikis & Stonis, 2013 [13] (p. 102).

**Figure 16:**
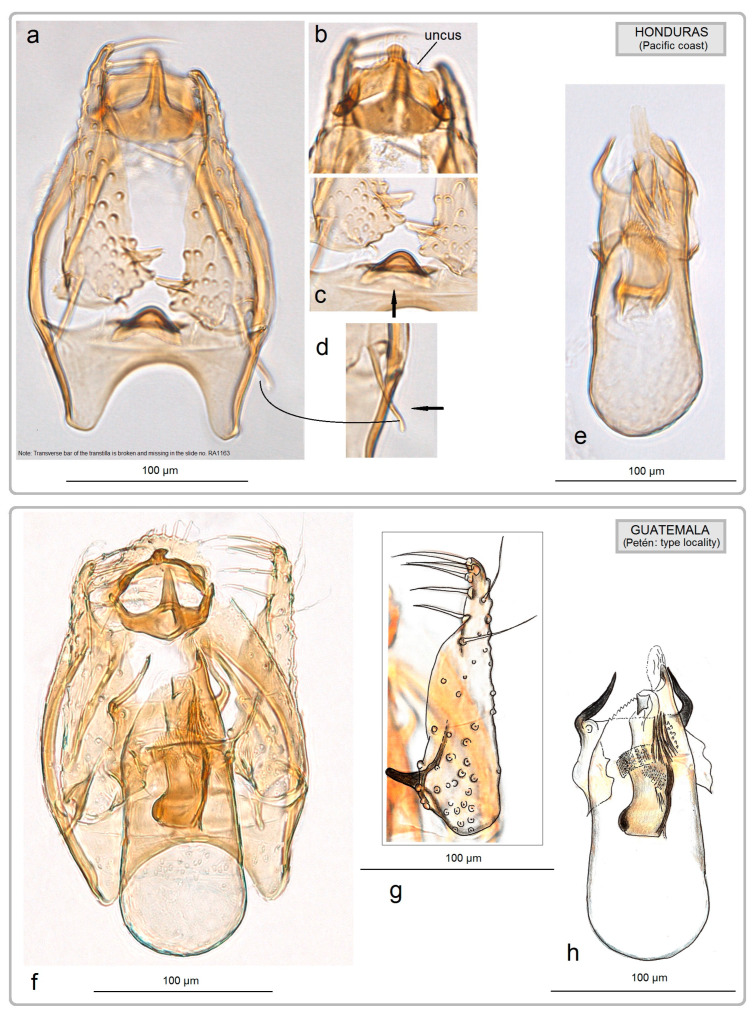
Comparison of the male genitalia of *Acalyptris basicornis* Remeikis & Stonis, a specimen discovered in the tropical dry forests, with the holotype from Guatemala: (**a**–**e**) genitalia slide no. RA1163, Pacific coast of Honduras (MfN); (**f**–**h**) holotype, genitalia slide no. RA478, Petén, Guatemala (after Stonis et al. [13]) (ZMUC).

**General data.** The host plant is unknown. The forewing length is 1.7 mm; the wingspan is 3.7 mm (n = 1 ♂) (Figure 3d). The species was originally described from a single specimen collected at a light trap in a moist tropical forest in Guatemala [13]. Based on the dissection and examination of a single specimen collected in the tropical dry forest (Figure 16a–e), we now report *A. basicornis* from Honduras for the first time, establishing a new distribution record.

**Remarks.** Our examination of *A. basicornis* collected in the tropical dry forests of Honduras did not indicated significant differences in the morphology of the male genitalia (Figure 16). However, the sublateral processes of the transtilla and the lateral lobes of the vinculum were found to be slightly longer than those of the holotype of *A. basicornis* from Guatemala. No barcodes were obtained for this species either previously or during the current research.

**Material examined.** 1 ♂, HONDURAS, the Pacific, Isla del Tigre, Amapala, Playa Grande, approx. 40 m, 13°16′32.3′′ N 87°39′37.5′′ W, 13–14 March 2023, leg. J.R. Stonis, genitalia slide no. RA1163♂ (MfN).

### 3.2. Molecular Considerations

*Acalyptris* Meyrick is the second largest genus of Nepticulidae in the Western Hemisphere (or so-called New World). In 2020, the previously known 56 species of this region were grouped into nine informal units, or species groups [20]. In order to find out which groups the species from the tropical dry forests of Honduras belong to, along with morphological characteristics, the investigation of the molecular aspects of these minute grey moths was particularly important. 

**Figure 17:**
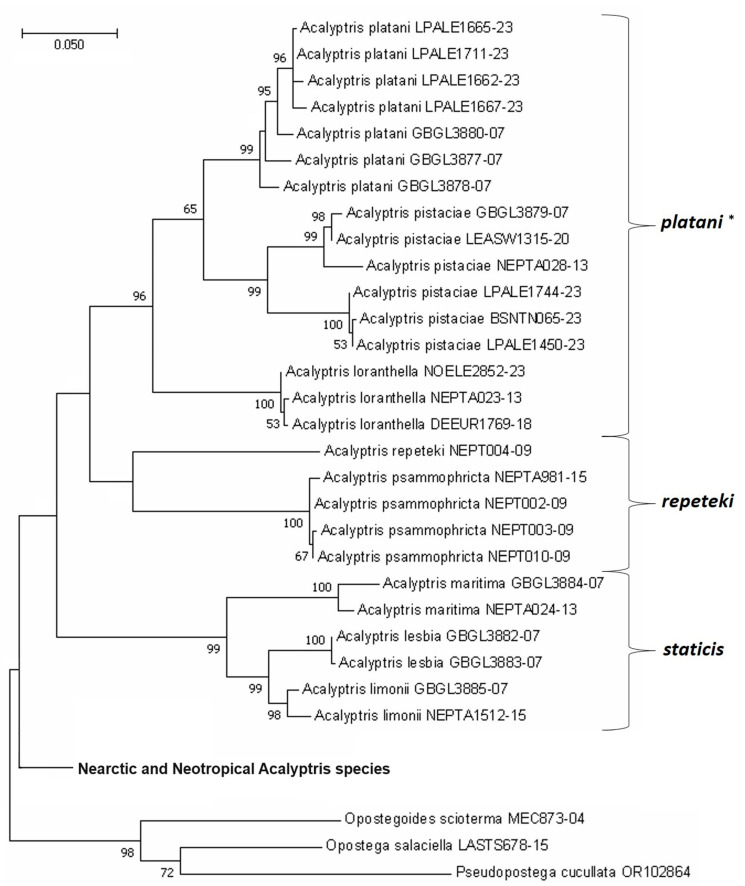
The Maximum Likelihood phylogenetic tree of the Palaearctic *Acalyptris* reconstructed based on the 657 bp-long mtDNA CO1-5′ sequences. The divergence was calculated using the GTR + G + I model. The percentages of replicate trees in which the associated taxa clustered together in the bootstrap test (10,000 replicates) are shown next to the branches (bootstrap values below 50 are not provided). Three Opostegidae species were included as an outgroup (*—species groups).

Consequently, we successfully sequenced eight specimens, including *Acalyptris lascuevella* for the first time, and all three new species, *A. podenasi* sp. nov., *A. palpiformis* sp. nov., and *A. tortoris* sp. nov. They were included in the molecular phylogenetic analyses using three methods (NJ, ML, and Bayesian inference) and different outgroups from the Opostegidae and Tischeriidae families. Unfortunately, none of them yielded a fully resolved tree, especially in the Bayesian analysis. The best outcomes still had doubtful dichotomies and low bootstrap support values in many cases. It seems that partial sequences of the mtDNA CO1 gene are not useful for discrimination of the Neotropical *Acalyptris*, unlike in the majority of *Acalyptris* species from Europe, elsewhere in the Mediterranean, and in Asia, where the topology of species was consistent (e.g., Figure 17) regardless of the method and outgroup. On the other hand, American *Acalyptris* are not only speciose but also very diverse in morphological characteristics. Moreover, their feeding preferences (host plants) are largely unknown but they may be the reason for their molecular peculiarities. It is possible that the applied NJ, ML, and Bayesian analyses simply do not work for the Neotropical *Acalyptris*, as is common in cases where the rate of evolution is extremely high [40].

**Figure 18:**
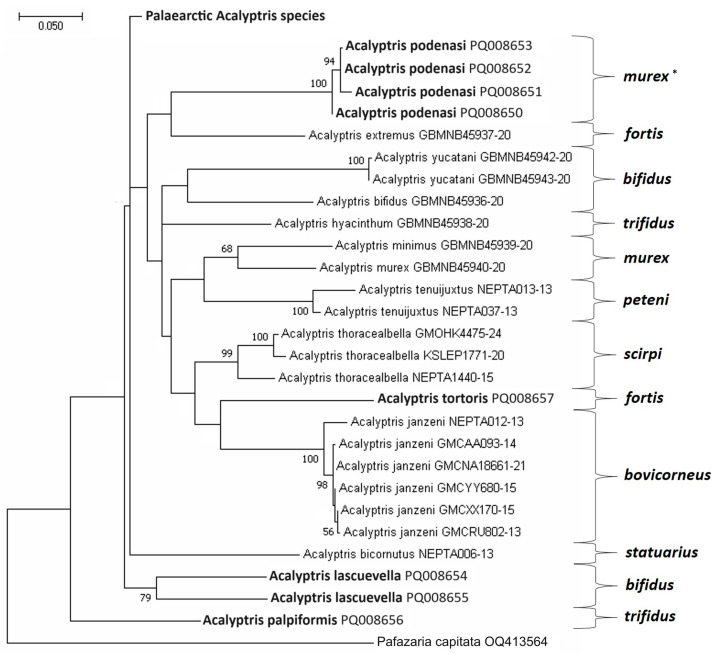
The Maximum Likelihood phylogenetic tree of the Nearctic and Neotropical *Acalyptris* reconstructed based on the 657 bp-long mtDNA CO1-5′ sequences. The divergence was calculated using the GTR + G + I model. The percentages of replicate trees in which the associated taxa clustered together in the bootstrap test (10,000 replicates) are shown next to the branches (bootstrap values below 50 are not provided). Tischeriidae sp. 1 was included as an outgroup (*—species groups).

**Figure 19:**
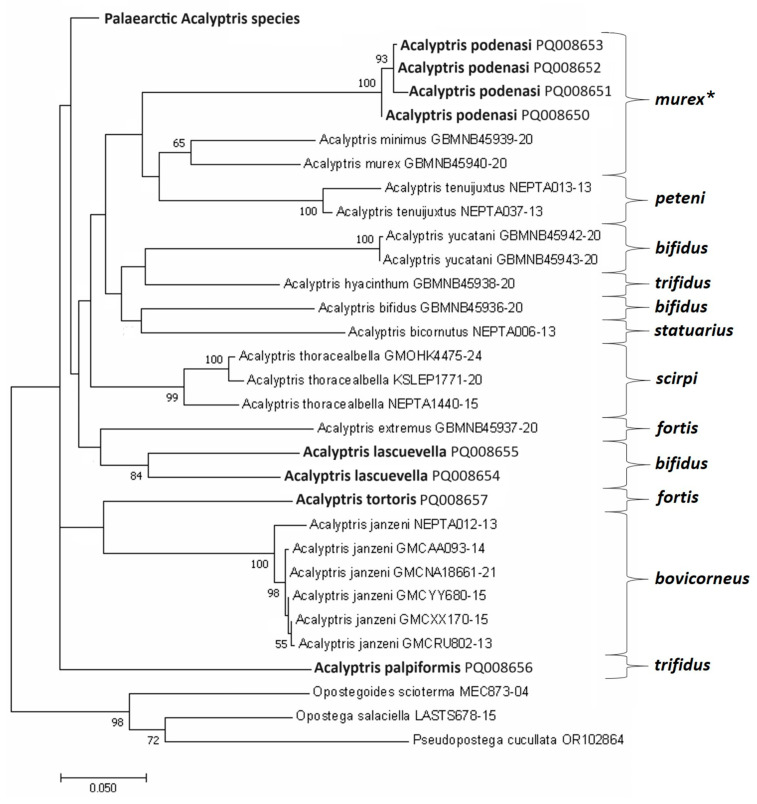
The Maximum Likelihood phylogenetic tree of the Nearctic and Neotropical *Acalyptris* reconstructed based on the 657 bp-long mtDNA CO1-5′ sequences. The divergence was calculated using the GTR + G + I model. The percentages of replicate trees in which the associated taxa clustered together in the bootstrap test (10,000 replicates) are shown next to the branches (bootstrap values below 50 are not provided). Three Opostegidae species were included as an outgroup (*—species groups).

Nevertheless, the majority of the constructed molecular trees supported the uniqueness of the previously designated species groups [20], which, with few exceptions, appeared as separate phylogenetic clades. In our analysis, *A. podenasi* sp. nov. often clustered with the *A. fortis* group (Figure 18) or, more frequently, with the clade of two groups: *murex* + *peteni* (Figure 19). This did not contradict the morphological characteristics of *A. podenasi* sp. nov.: in the female genitalia, this species shows similarities with the *A. murex* group, while in the male genitalia, it somewhat resembles a representative of the *A. peteni* group, *A. caribbicus* Diškus & Stonis, a *Lantana* feeder from Belize [13]. Despite *A. podenasi* sp. nov. being quite unique morphologically, we attributed this new species to the *A. murex* group but as a satellite (not core) species of the latter group.

*A. palpiformis* sp. nov., unexpectedly, provided the most intriguing case: its mtDNA CO1-5′ sequence appeared to be unique, often with a basal position in most of our molecular trees (e.g., Figure 18). It should be noted that in the male genitalia morphology, *A. palpiformis* sp. nov. is also characterized by a strikingly atypical uncus, previously unknown in *Acalyptris*. Nevertheless, in some other trees, *A. palpiformis* sp. nov. clustered with the *A. statuarius* group. The possibility of such a close relationship seems unlikely because it contradicts the morphological data. Consequently, we refrained from designating a new species group for *A. palpiformis* sp. nov. and for practical (diagnostic) reasons attributed it to the *A. trifidus* group, as in the male genitalia, all species of the *A. trifidus* group are relatively easily diagnosed by the uncus with three processes (or lobes), as is the case with *A. palpiformis* sp. nov.

Based on the heavily armed valva in the male genitalia, *A. tortoris* sp. nov. is classified within the *A. fortis* group. However, in most constructed trees, *A. tortoris* sp. nov. appeared as a sister clade to *A. janzeni* of the *A. bovicorneus* group (Figure 18 and Figure 19). This association has no support from the morphological characteristics because species in the *A. bovicorneus* group lack a transtilla, and their valvae are without spine-like processes. Even if *A. janzeni*’s current placement in the *A. bovicorneus* group is incorrect, *A. tortoris* sp. nov. does not resemble *A. bovicorneus* morphologically.

*A. lascuevella* was recently attributed to the *A. bifidus* group solely based on morphological similarity with other species possessing a two-lobed pseuduncus [20]. However, the first-ever molecular analysis did not confirm the position of *A. lascuevella* within the *A. bifidus* group. On the contrary, this species either remained unresolved or grouped with different groups (e.g., *A. fortis* (Figure 19)) without any clear prevalence. Morphologically, the species is easily diagnosed but exhibits significant variation in the inner processes of the valva and juxta in the male genitalia, as well as some variation in the forewing pattern. No correlation among these variable characters was found (sometimes, within the same specimen, the left side of the male genitalia looked different compared to the right side). Furthermore, in all phylogenetic trees, *A. lascuevella* sequences consistently exhibited an unusual, deep dichotomy, as for a single species (Figure 18 and Figure 19): the difference between these sequences reached as much as 11.24% ± 1.73, while the intraspecific divergence of the other most genetically variable *Acalyptris* species, *A. thoracealbella*, was at most 5.27% ± 1.08. Therefore, we report the variable nature of *A. lascuevella* both in external characteristics and in the male genitalia (see Remarks in the species section), as well as at the molecular level. It is also worth mentioning the peculiarities in the geographical distribution of this species: *A. lascuevella* was described 2.5 decades ago from the moist tropical forests of Belize, thereafter discovered on the Pacific coast of Mexico. Now, it is surprisingly found abundantly in the tropical dry forests of Honduras during the peak of the dry season. 

In conclusion, all species detected in the tropical dry forests and reviewed in our study appeared to be molecularly highly distinctive and quite specific, particularly in the case of the rather unique *A. palpiformis* sp. nov.

## 4. Discussion

**Taxonomic Affiliation and Morphological Peculiarities.** During the study, five species of pygmy moths were precisely and reliably identified, all belonging to the genus *Acalyptris* Meyrick. At the time of initial publication, the genus described by Edward Meyrick [39] was erroneously noted as having very small eye-caps (scapes), while the genus name “*Acalyptris*” itself means “without eye-caps”. However, species within the genus A*calyptris* are characterized by eye-caps, and all species found by us in the tropical dry forests not only have unusually large heads but also unusually large eye-caps, notably in the case of *A. podenasi* sp. nov. Most Neotropical *Acalyptris* species are typically creamy-yellow, not gray in color. Based on the external characteristics of adult specimens, all of these dry forest species are grey in color and are very similar to each other, differing only slightly. Therefore, identifying species based solely on external characteristics can be challenging or even impossible in cases of worn or variable individuals, typically indicating close species relationships. In contrast, an examination of the genital structures (and molecular studies) revealed that all species found in the study area are not only distinctly different from each other but also belong to different species groups (i.e., they are not closely related).

**Extremely small, minute moths.** All species currently detected in the tropical dry forests of Honduras are minuscule. *A. podenasi* sp. nov. and *A. tortoris* sp. nov. have forewing lengths ranging from 1.3 to 1.5 mm, *A. lascuevella* has a forewing length of 1.5–1.7 mm, *A. palpiformis* sp. nov. has a forewing length of 1.5–1.8 mm, and *A. basicornutus* has a forewing length of 1.7 mm. Overall, the wingspans of these species range from 3 to 4 mm. According to the size categories of adults provided by the paper “What are the smallest moths (Lepidoptera) in the world?” [3], all species reviewed in our article fall into the category of “extremely small,” with wingspans ranging from 3 to 4 mm. Based on previous calculations [3], extremely small species represent only about 12% of the global Nepticulidae fauna. Moreover, *A. podenasi* sp. nov. and *A. tortoris* sp. nov., with forewing lengths starting from 1.3 mm, are apparently among the smallest moths in the world. This obvious small size of the Nepticulidae in the tropical dry forests, along with their striking resemblance, characterizes the local Nepticulidae fauna as quite specific.

**Atypical Characters.** In the context of morphology, atypical characters refer to features that deviate from the common or expected characteristics of a species or higher taxon. These characters may be unusual, rare, or significantly different from the norm and can provide important insights into variations within a taxon, evolutionary adaptations, or environmental influences. Atypical characters have occasionally been reported in our research on the Neotropical Tischeriidae, another leaf-mining family of micromoths (citations). We believe that these discoveries can sometimes reveal new information about the biology, ecology, or evolutionary history of the organisms in question.

*Unusually Large Cells of Signum.* The structure of the signum varies in different ways, from a signum with a wide external body surrounding the inner cells found in nearly half of the Neotropical *Acalytris* (see [20]) to a very thin one observed in all Palaearctic (see [6,7,41] and Afrotropical species [5]. Moreover, the cells of the signum may also differ in size, from very small to relatively large. The study of females of *Acalyptris podenasi* sp. nov. from the tropical dry forests revealed unusually large cells of the signum, reaching up to 37–40 µm in width. Such large cells of the signum were documented for the first time, though their role is still unknown.

*Specialized Complex Uncus.* A type of uncus where this sclerite is divided into the main dorsal body with two lateral palp-like processes and the ventral lobe (ventral body) connected with the main body by thickened lateral arms was found. The diversity of the uncus in *Acalyptris* species can be found in the recent review of the genus of the Americas [20]. A type of uncus where the sclerite is divided dorsoventrally was found for the first time in the examination of *Acalyptris palpiformis* sp. nov. from the tropical dry forests.

*Asymmetry of Valvae.* Usually, Nepticulidae, including species from the genus Acalyptris, exhibit symmetry in the male genitalia. The examination of *A. tortoris* sp. nov. from the tropical dry forest habitats revealed asymmetry in the valva: in the ventral view, the left valva had a distinctive median spine (a short spine-like process) in all examined specimens, while the right valva had no spine-like process. Moreover, the right valva usually had a slightly or strongly dentate inner surface. Interestingly, *A. tortoris* sp. nov. was characterized by us as a species with very armed male genitalia possessing larger basal processes and many large, pointed carinae of the phallus. Additionally, the female of *A. tortoris* sp. nov. also has a highly specialized, complex vaginal sclerite, which is distinctly asymmetrical.

*Presence of Unique Ventral Plate of the Ovipositor.* During the examination of the female genitalia of *Acalyptris tortoris* sp. nov. from the tropical dry forest habitats, we possibly detected a novel character: a thickened ventral plate on the ovipositor ventrum. The function of such a thickened ventral plate remains unknown. We hypothesize that this may be related to oviposition on a specific host plant or, more likely, to a sensory lock-and-key mechanism that limits gene flow between species.

**Ecological Issues.** Nepticulidae are primarily known as leaf miners, with occasional occurrences on other plant parts. Despite accurately identifying the taxonomic affiliation of the species studied, a significant question arises regarding their presence during the dry season. Particularly at its peak, when extremely hot weather prevails and causes the complete drying of grass cover and the shedding of leaves from most trees, why do we observe such abundant findings of Nepticulidae adults?

It should be noted that moths abundantly attracted to our light traps from early February to late March (or early April) were mostly fresh and not worn, suggesting they had been alive shortly before capture. Given this observation, we ponder what these females might lay eggs upon, considering that most trees were leafless and the grass cover completely dry during our sampling.

Could it be possible that the *Acalyptris* species found in tropical dry forests during the dry season are miners of plant bark rather than leaves? However, there are currently no data to support this hypothesis, so the answer to this question (as well as many others) will need to be found in the future.

**Astonishing Abundance.** The phenomenon of the great abundance of these tiny grey nepticulid moths in the tropical dry forests during the peak of the dry season is another interesting question. In general, pygmy moths are not very common at light traps. For instance, in the tropical moist (humid) forests of Honduras, we averaged 6–10 specimens per night collection session or none almost every other night. In the tropical dry forests, however, pygmy moths were always abundant, and during night collection sessions at the peak of the dry season, we often observed over a hundred arrivals at the light trap. Sometimes, the arrivals were so numerous that it was physically impossible to sample or count them all. It should be noted that in both the wet tropical forests and the tropical dry forests of Honduras, we used the same light collecting techniques, the same lanterns, and the same duration of night samplings. However, there was one slight difference: the average temperature during a night collection session in the tropical dry forest in February–March was +30 °C (with a maximum reaching up to +32 °C), while in the tropical moist (humid) forests, the average temperature during a night collection session in April 2023 and February–April 2024 was +28 °C (with a maximum of +30 °C). This temperature difference could partially account for the difference in the abundance of arrivals. Nevertheless, it still cannot fully explain the great abundance of pygmy moths in the tropical dry forests during the peak of the dry season.

**Revealing an Unexpected Phenomenon.** The tropical dry forests are crucial for global biodiversity and are among the world’s most endangered and rapidly disappearing biomes [27,42]. Despite their significance, they have been entirely overlooked in studies of minuscule, phylogenetically primitive leaf-mining Lepidoptera. Our own experience highlights this gap: despite 25 years of studying leaf-mining Lepidoptera in tropical environments, we had never considered setting a trap in a completely dry deciduous forest, particularly during the peak of the dry season when trees are bare and even the grass cover is entirely dried out. Consequently, the discovery of adults of leaf-mining micromoths (and in surprising abundance) in such a leafless forest during the peak of the prolonged dry season represents an unexpected and previously unknown phenomenon.

The manuscript’s primary importance lies not just in the “simple” description of new species but in uncovering their taxonomic, morphological, and molecular significance. We found that the detected species are unique and indicative of the previously unstudied dry forests as a distinct ecosystem.

We hope that our paper will now encourage Lepidoptera taxonomists to explore micromoths in other tropical dry forests, which, while limited in distribution, have worldwide importance. It would be fascinating to uncover how much is still unknown about tropical dry forests and to contribute to the effort of protecting this unique biome.

**Future Prospects.** Although the studies were conducted in seven different tropical dry forest locations for a considerable period (during the peak of the dry season from early February to the end of March), we believe that many more externally similar but taxonomically distinct *Acalyptris* species may be discovered on the Pacific coast in the future, especially given the region’s considerable habitat diversity. A few other species of the *Acalyptris* genus were sparsely found during the study, though they were not included in this publication as they did not match the publication’s theme and will be published later.

## Data Availability

The molecular data presented in this study can be found in online repositories (GenBank). Open access depositories of the collection material (physical specimens and their genitalia mounts) are listed in the article.
